# Retraction: Comparison of triglyceride-glucose index and triglyceride-glucose related indexes in predicting cardiovascular disease incidence among populations with cardiovascular-kidney-metabolic syndrome stages 0-3: a nationwide prospective cohort study

**DOI:** 10.3389/fendo.2026.1912913

**Published:** 2026-06-26

**Authors:** Frontiers Editorial Office

The journal retracts the 22 August 2025 article cited above.

Following concerns regarding the originality of the article, an investigation was conducted in accordance with Frontiers’ policies. It was found that the complaint was valid and that the article should be retracted, because of an unacceptable level of similarity *in the figures and tables* to an article published by Springer Nature:

Hong, J., Zhang, R., Tang, H. et al. Comparison of triglyceride glucose index and modified triglyceride glucose indices in predicting cardiovascular diseases incidence among populations with cardiovascular-kidney-metabolic syndrome stages 0–3: a nationwide prospective cohort study. Cardiovasc Diabetol 24, 98 (2025). https://doi.org/10.1186/s12933-025-02662-3

This retraction was approved by the Field Chief Editor of Frontiers of Endocrinology and the Chief Executive Editor of Frontiers. The authors cooperated with this investigation and agree to this retraction. The authors agree to correspondence regarding this retraction.

Frontiers would like to thank Jame Yu for contacting the journal regarding the published article. Frontiers would also like to thank the users on PubPeer for bringing the published article to our attention.

